# Resveratrol potently reduces prostaglandin E_2 _production and free radical formation in lipopolysaccharide-activated primary rat microglia

**DOI:** 10.1186/1742-2094-4-25

**Published:** 2007-10-10

**Authors:** Eduardo Candelario-Jalil, Antonio C Pinheiro de Oliveira, Sybille Gräf, Harsharan S Bhatia, Michael Hüll, Eduardo Muñoz, Bernd L Fiebich

**Affiliations:** 1 Neurochemistry Research Group, Department of Psychiatry, University of Freiburg Medical School, Hauptstrasse 5, D-79104 Freiburg, Germany; 2 Department of Neurology, University of New Mexico Health Sciences Center, Albuquerque, NM 87131, USA; 3 Center for Geriatrics and Gerontology, University of Freiburg Medical School, Lehenerstrasse 88, D-79106 Freiburg, Germany; 4 Departamento de Biología Celular, Fisiología e Inmunología. Universidad de Córdoba, Avda Menéndez Pidal s/n. 14004, Córdoba, Spain; 5 VivaCell Biotechnology GmbH, Ferdinand-Porsche-Str. 5, D-79211 Denzlingen, Germany

## Abstract

**Background:**

Neuroinflammatory responses are triggered by diverse ethiologies and can provide either beneficial or harmful results. Microglial cells are the major cell type involved in neuroinflammation, releasing several mediators, which contribute to the neuronal demise in several diseases including cerebral ischemia and neurodegenerative disorders. Attenuation of microglial activation has been shown to confer protection against different types of brain injury. Recent evidence suggests that resveratrol has anti-inflammatory and potent antioxidant properties. It has been also shown that resveratrol is a potent inhibitor of cyclooxygenase (COX)-1 activity. Previous findings have demonstrated that this compound is able to reduce neuronal injury in different models, both *in vitro *and *in vivo*. The aim of this study was to examine whether resveratrol is able to reduce prostaglandin E_2 _(PGE_2_) and 8-*iso*-prostaglandin F_2α _(8-*iso*-PGF_2α_) production by lipopolysaccharide (LPS)-activated primary rat microglia.

**Methods:**

Primary microglial cell cultures were prepared from cerebral cortices of neonatal rats. Microglial cells were stimulated with 10 ng/ml of LPS in the presence or absence of different concentrations of resveratrol (1–50 μM). After 24 h incubation, culture media were collected to measure the production of PGE_2 _and 8-*iso*-PGF_2α _using enzyme immunoassays. Protein levels of COX-1, COX-2 and microsomal prostaglandin E synthase-1 (mPGES-1) were studied by Western blotting after 24 h of incubation with LPS. Expression of mPGES-1 at the mRNA level was investigated using reverse transcription-polymerase chain reaction (RT-PCR) analysis.

**Results:**

Our results indicate that resveratrol potently reduced LPS-induced PGE_2 _synthesis and the formation of 8-*iso*-PGF_2α_, a measure of free radical production. Interestingly, resveratrol dose-dependently reduced the expression (mRNA and protein) of mPGES-1, which is a key enzyme responsible for the synthesis of PGE_2 _by activated microglia, whereas resveratrol did not affect the expression of COX-2. Resveratrol is therefore the first known inhibitor which specifically prevents mPGES-1 expression without affecting COX-2 levels. Another important observation of the present study is that other COX-1 selective inhibitors (SC-560 and Valeroyl Salicylate) potently reduced PGE_2 _and 8-*iso*-PGF_2α _production by LPS-activated microglia.

**Conclusion:**

These findings suggest that the naturally occurring polyphenol resveratrol is able to reduce microglial activation, an effect that might help to explain its neuroprotective effects in several *in vivo *models of brain injury.

## Background

Resveratrol (*trans*-3,5,4'-trihydroxystilbene) is a polyphenolic compound present in relatively large amounts in grapes and red wine. In smaller quantities, resveratrol is also present in almost 70 plant species, where it has been found to act as an anti-fungicide and confer disease resistance in the plant kingdom [1]. Recently, this natural compound has received a great deal of attention due to its ability to serve as a potent antioxidant [2]. In addition, resveratrol has been proven to possess anti-inflammatory, immunomodulatory, chemopreventive, neuroprotective, and cardioprotective properties [3-10].

One of the most interesting properties of resveratrol is its ability to confer potent neuroprotection in several models of brain injury, both *in vitro *[10-12] and *in vivo *[7,8,13,14]. Resveratrol readily crosses the intact blood-brain barrier as demonstrated in previous studies [7,15]. There is much evidence from recent studies, which indicate that ischemic brain injury is potently reduced in resveratrol-treated animals. The first report suggesting that cerebral infarction is significantly diminished by systemic administration of resveratrol comes from Huang et al. [13], using an *in vivo *model of focal cerebral ischemia in rats. In another study, resveratrol increased the number of CA1 hippocampal neurons surviving a global cerebral ischemic insult [7]. Resveratrol not only reduced neuronal death but also reduced the number of reactive astrocytes and activated microglial cells [7]. The free radical scavenging ability seems to underlie the efficacy of resveratrol against neuronal demise in cerebral ischemia, as suggested in a recent study [16].

In order to explain at the molecular level the mechanisms responsible for resveratrol neuroprotection under ischemic conditions, *in vitro *models involving neuronal cultures as well as hippocampal slices subjected to oxygen-glucose deprivation have been employed. Nitric oxide-related toxicity to cultured hippocampal neurons was dramatically inhibited by resveratrol through a mechanism that seems to be at least partially related to its antioxidant effect [11]. Similarly, resveratrol attenuated cell death in organotypic hippocampal slice cultures exposed to oxygen-glucose deprivation through activation of the phosphoinositide-3-kinase (PI3-K)/Akt pathway [17].

The neuroprotective effect of resveratrol is not only restricted to cerebral ischemia. This natural compound also reduced oxidative stress and lesion volume in a model of traumatic brain injury [18] and spinal cord injury [19,20] in rats. Furthermore, resveratrol protected against excitotoxicity induced by kainic acid [8], and oxidative stress and behavioral changes in a rat model of Huntington's disease [21]. In addition, it has been recently demonstrated that resveratrol promotes intracellular degradation of amyloid β peptide via a mechanism that involves the proteasome [22].

Although mounting evidence convincingly demonstrates the potential of resveratrol to provide significant protection against different types of brain injury, the exact molecular mechanisms responsible for these beneficial effects are not fully elucidated. Its antioxidant ability alone can not give an explanation to the wide array of pharmacological properties of this compound.

Microglial cells are important protagonists in the cascade of events leading to tissue injury following neurodegeneration and other types of cerebral damage [23-28]. Very few studies have investigated the effects of resveratrol on microglial activation during neuroinflammation. In an earlier study, resveratrol was found to produce a potent suppressive effect on tumor necrosis factor α (TNFα) and nitric oxide production induced by lipopolysaccharide (LPS) in the mouse microglial cell line N9 [29]. These effects are mediated through inhibition of nuclear factor κB (NF-κB) activation and p38 MAPK phosphorylation [29].

Based on these previous reports, the present study was undertaken to investigate the effects of resveratrol on neuroinflammatory events associated to the production of prostaglandin E_2 _and reactive oxygen species (ROS) in activated primary microglial cells. Our findings indicate for the first time that resveratrol is a potent inhibitor of PGE_2 _production in microglia through a mechanism that involves reduction in the expression of the key enzyme involved in PGE_2 _formation, namely, microsomal prostaglandin E synthase-1 (mPGES-1). Direct inhibitory effects of resveratrol on cyclooxygenase activity as well as reduction in 8-isoprostane formation are other beneficial effects that contribute to the potent attenuation of microglial activation, and possibly to the neuroprotective efficacy of this natural compound in different models of brain injury.

## Methods

### Reagents and Antibodies

*Trans*-resveratrol, Trolox C, α-tocopherol, and LPS (from *Salmonella typhimurium*) were obtained from Sigma-Aldrich (Taufkirchen, Germany). LPS was resuspended in sterile phosphate buffered saline (PBS; Cell Concepts, Umkirch, Germany) as 5 mg/ml stock, and was used at a final concentration of 10 ng/ml in the culture. The COX-1 inhibitors SC-560 and Valeroyl salicylate (VAS) were obtained from Cayman Chemical Co. (Ann Arbor, USA). Resveratrol, Trolox C, and α-tocopherol were dissolved in absolute ethanol. SC-560 and VAS were dissolved in DMSO and water, respectively. Solvent concentration in the culture media was maintained at less than 0.1%. All agents, used at the given concentrations, do not affect the viability of the cells as observed through a luminescent kit (Promega, Madison, WI, USA), which measures metabolic ATP levels (data not shown). Antibodies against COX-1 (M-20) and COX-2 (M-19) were from Santa Cruz Biotechnology (Santa Cruz, CA, USA). Rabbit polyclonal antibody against mPGES-1 was obtained from Cayman Chemical Co. (Ann Arbor, MI, USA), while the antibody against actin was from Sigma (Saint Louis, MO, USA).

### Primary rat microglial cell culture

Primary mixed glial cell cultures were established from cerebral cortices of one-day neonatal Sprague-Dawley rats as described in details in our previous reports [30-34]. Briefly, forebrains were minced and gently dissociated by repeated pipetting in PBS and filtered through a 70-μm cell strainer (Falcon). Cells were collected by centrifugation (1000 rpm, 10 min), resuspended in Dulbecco's modified Eagle's medium (DMEM) containing 10% fetal calf serum (Biochrom AG, Berlin, Germany) and antibiotics (40 U/ml penicillin and 40 μg/ml streptomycin, both from PAA Laboratories, Linz, Austria), and cultured on 10-cm cell culture dishes (5 × 10^5 ^cells/plate, Falcon) in 5% CO_2 _at 37°C. Medium was prepared taking extreme care to avoid LPS contamination [35]. Floating microglia were harvested every week (between 2–7 week) and re-seeded into 75 cm^2 ^culture flask (for RNA extraction and Western blots) or 24-well plates (for 8-isoprostane and PGE_2 _estimation) to give pure microglial cultures. The following day, cultures were washed to remove non-adherent cells, and fresh medium was added. The purity of the microglial culture was >98% as previously determined by immunofluorescence and cytochemical analysis [35].

### Prostaglandin E_2 _(PGE_2_) Enzyme Immunoassay

Supernatants were harvested, centrifuged at 10,000 × g for 10 min and levels of PGE_2 _in the media were measured by enzyme immunoassay (EIA) (Assay Designs Inc., Ann Arbor, MI, USA; distributed by Biotrend, Cologne, Germany) according to the manufacturer's instructions. Standards from 39 to 2500 pg/ml were used. The sensitivity of this assay is 36.2 pg/ml.

### Cyclooxygenase Activity Assay

To determine any direct inhibitory effect of resveratrol on COX-1 and COX-2 enzymatic activity, an arachidonic acid assay was performed as described [36,37]. Briefly, primary rat microglial cells were plated in 24-well cell culture plates, and pre-incubated with LPS (10 ng/ml) for 24 h. Medium was then removed, and cells washed in serum-free medium. Resveratrol (5–50 μM) was added, and after 15 min pre-stimulation, 15 μM arachidonic acid was supplemented for another 15 min. Supernatants were then used for determination of PGE_2 _[36]. We also investigated the effects of resveratrol on microglial COX-1 enzymatic activity. Under unstimulated conditions, primary microglial cells only express the COX-1 isoform [34]. Thus, the COX-1 activity assay was conducted exactly as mentioned before without pre-incubation with LPS.

### RNA Isolation and Reverse Transcription-Polymerase Chain Reaction (RT-PCR)

Total RNA was extracted using the guanidine isothiocyanate method according to Chomczynski and Sacchi [38]. For RT-PCR, 2 μg of total RNA was reverse transcribed using Moloney Murine Leukemia Virus (M-MLV) reverse transcriptase (Promega, Mannheim, Germany), RNase Inhibitor rRNasin^® ^(Promega), dNTP master mix (Invitek, Berlin, Germany) and random hexamer primers (Promega). PCR was carried out using Taq DNA polymerase (Promega), dNTP master mix (Invitek, Berlin, Germany) and the following primers: rat microsomal prostaglandin E synthase-1, mPGES-1 (forward: 5'-ATG ACT TCC CTG GGT TTG GTG ATG GAG -3', reverse: 5'- ACA GAT GGT GGG CCA CTT CCC AGA -3', annealing temperature 65°C, 35 cycles, amplicon size: 459 bp). Primers for rat COX-2 were as follows: forward, 5'-TGC GAT GCT CTT CCG AGC TGT GCT-3' and reverse, 5'- TCA GGA AGT TCC TTA TTT CCT TTC-3', annealing temperature 55°C, 35 cycles, amplicon size: 479 bp). Primers used for amplifying a fragment of 426 bp from rat COX-1 mRNA were: forward, 5'-CGG CCT CGA CCA CTA CCA ATG -3' and reverse, 5'- TGC GGG GCG GGA ATG AAC T-3', annealing temperature 60°C, 30 cycles. Equal equilibration was determined using rat β-actin primers (forward: 5'- ATG GAT GAC GAT ATC GCT -3', reverse: 5'- ATG AGG TAG TCT GTC AGG T -3', 48°C, 30 cycles, product length: 569 bp) or primers for S12 from rat (forward: 5'- ACG TCA ACA CTG CTC TAC A -3', reverse: 5'- CTT TGC CAT AGT CCT TAA C -3', 56°C, 30 cycles, product length: 312 bp). PCR products were separated electrophoretically on a 2% agarose gel. Potential contamination by genomic DNA was controlled by omitting reverse transcriptase and using primers for the housekeeping genes (β-actin or S12) in the subsequent PCR amplification. Only RNA samples showing no bands after this procedure were used for further investigation. Primers were designed using the Primer3 software developed by the Whitehead Institute for Biomedical Research [39], and synthesized through an in-house facility (Dr. Gabor Igloi, Institute for Biology III, Freiburg, Germany). PCR analysis was performed after 4 h of stimulation with LPS.

### Western blot analysis

For COX-1, COX-2 and mPGES-1 immunoblotting, microglial cells were left untreated or treated with LPS (10 ng/ml) in the presence or absence of resveratrol (1–50 μM) for 24 h. Cells were washed with phosphate buffered saline (PBS) and lysed in 1.3x SDS (sodium dodecyl sulfate)-containing sample buffer without DTT or bromophenol blue containing 100 μM orthovanadate [40]. Lysates were homogenized by repeated passage through a 26-gauge needle. Protein contents were measured using the bicinchoninic acid method (BCA protein determination kit from Pierce, distributed by KFC Chemikalien, Munich, Germany) according to the manufacturer's instructions. Bovine serum albumin (BSA, Sigma) was used as a standard. Before electrophoresis, bromophenol blue and DTT (final concentration, 10 mM) were added to the samples. For Western blotting, 60 μg of total protein from each sample were subjected to SDS-PAGE (polyacrylamide gel electrophoresis) under reducing conditions. Proteins were then transferred onto a polyvinylidene fluoride (PVDF) membrane (Millipore, Bedford, MA, USA) by semi-dry blotting. The membrane was blocked overnight at 4°C using Rotiblock (Roth, Karlsruhe, Germany) and for another hour at room temperature before incubation with the primary antibody. Primary antibodies were goat anti-COX-2, goat anti-COX-1 and rabbit anti-mPGES-1. Primary antibodies were diluted 1:500 in Tris-buffered saline (TBS) containing 0.1% Tween 20 (TBST) and 1% bovine serum albumin (BSA, Sigma). Membranes were incubated with the corresponding primary antibody for 2 h at room temperature. After extensive washing (three times for 15 min each in TBST), proteins were detected with horseradish peroxidase-coupled rabbit anti-goat IgG (Santa Cruz, 1:100,000 dilution) or goat anti-rabbit IgG (Amersham, 1:25,000 dilution) using chemiluminescence (ECL) reagents (Amersham Pharmacia Biotech, Freiburg, Germany). Quantification of the Western blots was performed using ScanPack 3.0 software (Biometra, Göttingen, Germany). Equal protein loading and transfer were assessed by subjection of each sample to a Western blot for actin (rabbit anti-actin IgG, diluted 1:5000). All western blot experiments were carried out at least three times.

### Determination of 8-*iso*-prostaglandin F_2α _(8-*iso*-PGF_2α_)

8-isoprostanes are formed in response to free radical attack on arachidonic acid on membrane phospholipids and are considered as a reliable and highly sensitive measure of free radical formation [41]. Microglial cells were pre-treated for 30 min with different concentrations of resveratrol, Trolox C, α-tocopherol, SC-560, or VAS (see Results and Figures). After 30 min pre-stimulation, cells were added 10 ng/ml LPS for 24 h. Control experiments consisted of cells treated only with the solvent of each compound without LPS. Supernatants were harvested and the levels of 8-*iso*-PGF_2α _(IUPAC nomenclature: 15-F_2t_-IsoP) were measured by an enzyme immunoassay according to the manufacturer's instructions (Cayman Chemicals, Ann Arbor, MI, USA). The standards were used in the range of 3.9 to 500 pg/ml (detection limit of 5 pg/ml).

### Data analysis

Data from at least 3 experiments were used for data analysis. Original data were converted into %-values of LPS control and mean ± S.E.M. were calculated. Values were compared using *t*-test (two groups) or one-way ANOVA with *post-hoc *Student-Newman-Keuls test (multiple comparisons).

## Results

**Figure 1:**
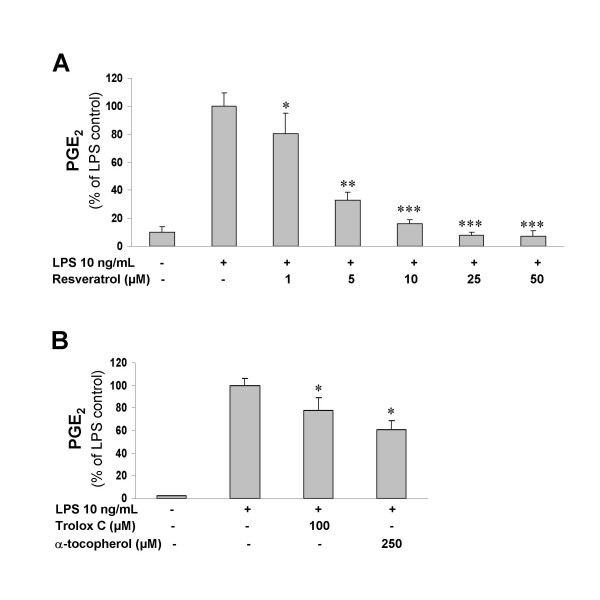
**A: **Resveratrol dose-dependently inhibits LPS-induced PGE_2 _production in primary rat microglial cells. **B: **Effects of Trolox C and α-tocopherol on PGE_2 _production by microglia challenged with LPS. The amounts of PGE_2 _in the culture medium were determined using an enzyme immunoassay after 24 h incubation with LPS. Substances were added 30 min before stimulating the cells with LPS. Each column and error bar represents the mean ± S.E.M. of 4 independent experiments. Asterisks indicate significant difference between the treatments. *p < 0.05, **p < 0.01 and ***p < 0.001 with respect to LPS control (One-way ANOVA followed by the Student-Newman-Keuls *post-hoc *test).

In our initial experiments, we investigated the effects of resveratrol on LPS-induced PGE_2 _production in microglia. As shown in Fig. 1A, resveratrol potently diminished PGE_2 _synthesis starting at 1 μM and showing a dose-dependent inhibitory effect between 1 and 10 μM. The higher doses of resveratrol tested in this study did not further decrease PGE_2 _formation, maintaining PGE_2 _at basal levels (not different from unstimulated control cells). Since resveratrol is a potent antioxidant compound, we decided to examine if other antioxidants were also able to modify PGE_2 _production by activated microglia. In contrast to resveratrol, the antioxidants α-tocopherol and its synthetic analogue Trolox C only slightly reduced PGE_2 _biosynthesis at relatively high doses (100 and 250 μM respectively; Fig. 1B). These antioxidant compounds were unable to reduce PGE_2 _formation when added to the microglial cultures at lower doses (data not shown).

**Figure 2:**
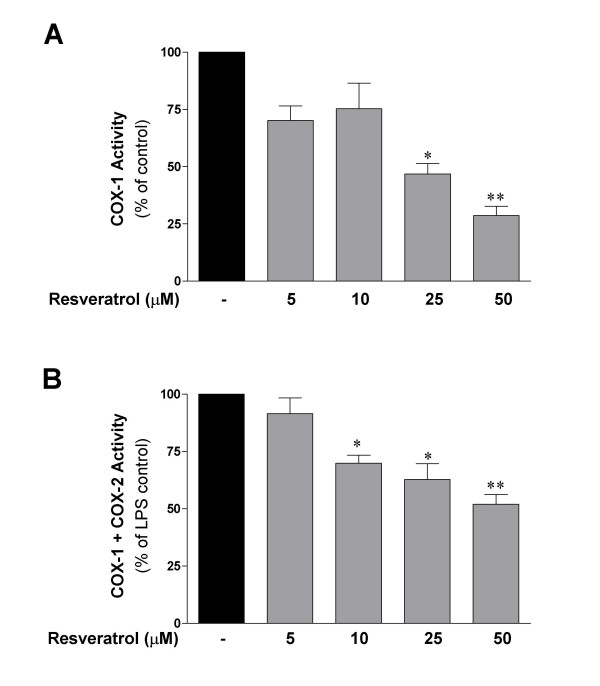
Cyclooxygenase enzymatic activity in microglial cells is inhibited by resveratrol treatment. For the COX-1 activity assay (**A**), cells were left untreated and different concentrations of resveratrol were added for 15 min. After this incubation time, 15 μM of arachidonic acid was added and PGE_2 _measured after 15 min. For total COX activity assay (COX-1 + COX-2), cells were either left untreated or were stimulated with LPS (10 ng/ml) for 24 h. After removal of medium, cells were treated with different concentrations of resveratrol for 30 min in absence or presence of 15 μM of arachidonic acid. PGE_2 _in the supernatants was measured by an enzyme immunoassay as described in Materials and Methods. Data are expressed as mean ± S.E.M. Statistical analysis was performed using one-way ANOVA followed by the Student-Newman-Keuls *post-hoc *test. *p < 0.05 and **p < 0.01 with respect to untreated control (without resveratrol).

These findings prompted us to investigate the ability of resveratrol to reduce COX enzymatic activity in activated microglia. Results from this experiment are shown in Fig. 2. Significant inhibition of COX-1 activity was observed when microglial cells were pre-incubated for 30 min with 25 and 50 μM of resveratrol (Fig. 2A). The effect of resveratrol on total COX activity (COX-1 + COX-2) was also investigated. In this *in vitro *assay, cells were pre-incubated with LPS for 24 h before resveratrol was added for 30 min, and PGE_2 _levels were measured in the supernatant. The addition of resveratrol produced a significant inhibition of COX activity starting at 10 μM and showing a 50% inhibition with the highest dose of 50 μM (Fig. 2B).

**Figure 3:**
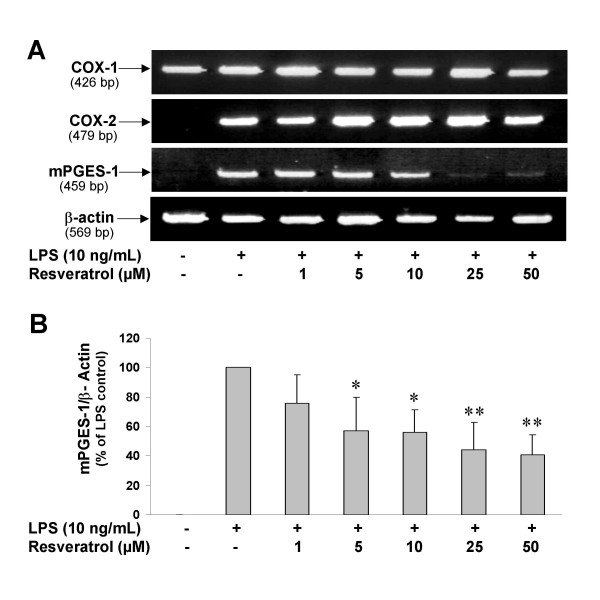
**A: **Representative photomicrographs showing RT-PCR products of COX-1, COX-2, mPGES-1, and β-actin mRNAs. Microglial cells were treated with LPS (10 ng/ml) in the absence or presence of different concentrations of resveratrol. The mRNA expression levels were tested for each condition. There is a constitutive COX-1 mRNA expression that is observed under all conditions. However, COX-2 and mPGES-1 are undetectable in untreated microglial cells, while their expression is dramatically increased in the presence of LPS. Resveratrol treatment significantly reduced mPGES-1, but not COX-2 expression. RT-PCR analysis was performed after 4 h of incubation with LPS. Resveratrol was added to the cultures 30 min before LPS. **B: **Semi-quantitative analysis of the effect of resveratrol on mPGES-1 expression. This analysis was done with results from 4 different RT-PCR experiments performed independently. Relative expression of rat β-actin was used for normalization. The amplicon size of the PCR product appears below the name of each transcript. *p < 0.05 and **p < 0.01 with respect to untreated LPS control.

**Figure 4:**
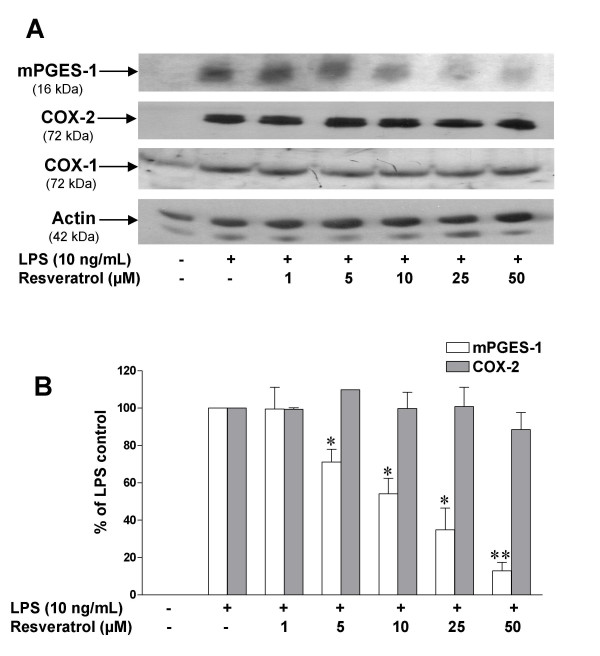
**A: **Immunoblot analysis of protein levels of COX-1, COX-2, mPGES-1, and actin in LPS-activated microglia treated with different concentrations of resveratrol (1–50 μM). Microglial protein extracts were prepared after 24 h of incubation with LPS, and subjected to SDS-PAGE followed by immunoblot analysis using specific antibodies for each protein. **B: **Quantitative densitometric analysis of mPGES-1 and COX-2 protein expression normalized to actin loading control. Resveratrol potently reduced mPGES-1 protein expression induced by LPS. *p < 0.05 and **p < 0.01 with respect to untreated LPS control. Bars represent mean ± S.E.M. of 4 independent experiments.

Since there are significant changes in gene expression in LPS-activated microglia, including a dramatic upregulation of the PGE_2 _synthesizing enzymes COX-2 and mPGES-1 [31,34,42], we decided to investigate the effects of resveratrol on the expression of these key enzymes responsible for PGE_2 _production in LPS-stimulated microglia, at both the mRNA and protein levels. RT-PCR analysis showed that control microglial cells do not express COX-2 or mPGES-1 mRNA under control conditions, but the expression of these enzymes is dramatically induced by addition of LPS to the cultures (Fig. 3A). Of great interest is our finding that resveratrol produced a significant reduction in the expression of mPGES-1 mRNA, without modifying COX-2 mRNA expression (Figs. 3A and 3B). Similar results were observed when investigating the effects of resveratrol on mPGES-1 protein levels as assessed by Western blotting (Fig. 4A). It is important to emphasize that resveratrol did not modified basal COX-1 or LPS-induced COX-2 expression in microglia. A densitometric analysis of protein levels of mPGES-1 and COX-2, and the effects of resveratrol are presented in Fig. 4B.

**Figure 5:**
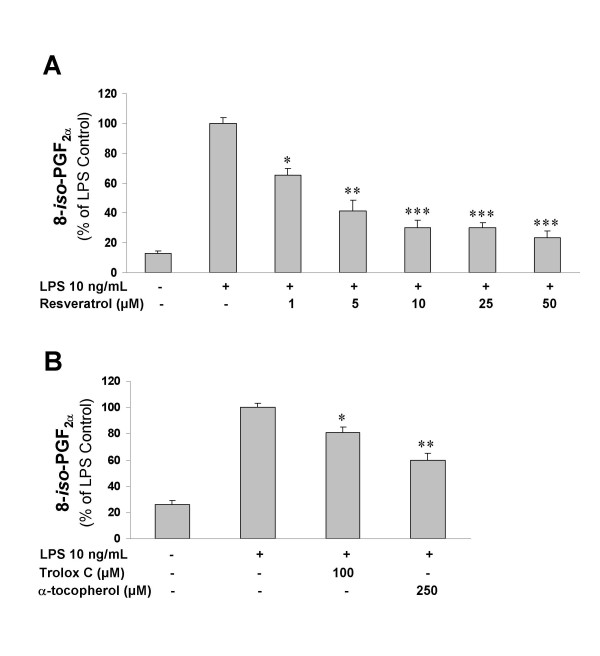
**A: **Effect of resveratrol on 8-*iso*-prostaglandin F_2α _(8-*iso*-PGF_2α_) production in response to 10 ng/ml LPS. 8-*iso*-PGF_2α _was determined in the culture medium 24 h following stimulation of microglial cells with LPS alone, or in combination with resveratrol at the given concentrations. **B: **Moderate reduction in LPS-mediated 8-*iso*-PGF_2α _formation by the antioxidants Trolox C and α-tocopherol. *p < 0.05, **p < 0.01 and ***p < 0.001 with respect to LPS alone. For both panels, histograms represent mean ± S.E.M. of 4 independent experiments. Statistical analysis was performed using one-way ANOVA followed by Student-Newman-Keuls *post-hoc *test.

Since resveratrol has been proven to have potent antioxidant properties, we aimed to determine the efficacy of this compound in reducing free radical production by activated microglia. LPS produced a very significant increase in the formation of 8-*iso*-PGF_2α_, a very sensitive marker of cellular free radical generation, as assessed by enzyme immunoassay (Fig. 5). More importantly, resveratrol potently reduced LPS-mediated formation of ROS, an effect that was seen at very low doses (starting at 1 μM). This reduction in 8-*iso*-PGF_2α _by resveratrol showed a concentration-dependent response between 1 and 10 μM (Fig. 5A), an effect also seen in our first experiment evaluating PGE_2 _levels (Fig. 1A). We then tested the ability of two well-known antioxidants, Trolox C and α-tocopherol, to reduce free radical formation in LPS-activated microglia. These antioxidants significantly reduced 8-*iso*-PGF_2α _as well, but not as potent as resveratrol (Fig. 5B).

**Figure 6:**
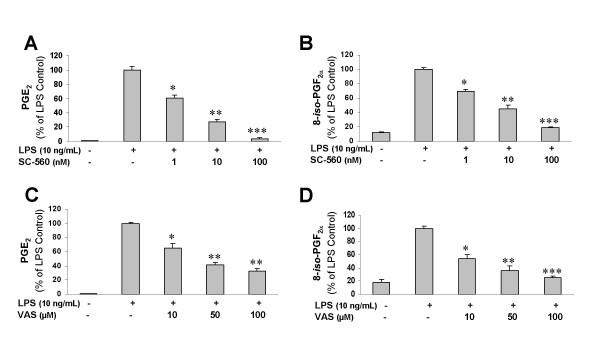
Potent inhibition of LPS-induced PGE_2 _(**A **and **C**) and 8-*iso*-PGF_2α _formation (**B **and **D**) by two highly selective COX-1 inhibitors (SC-560 and VAS). After 30 min pre-stimulation with the inhibitors, LPS (10 ng/mL) was added to the culture medium, and the amounts of PGE_2 _and 8-*iso*-PGF_2α _were determined using specific enzyme immunoassays after 24 h incubation with LPS. For all panels, histograms represent mean ± S.E.M. of 4 independent experiments. Statistical analysis was performed using one-way ANOVA followed by Student-Newman-Keuls *post-hoc *test. *p < 0.05, **p < 0.01 and ***p < 0.001 with respect to LPS control.

The ability of resveratrol to inhibit the peroxidase activity of COX-1 is a well-known pharmacological effect of this compound [43,44]. It was then very important to investigate the ability of other COX-1 inhibitors to reduce PGE_2 _and 8-*iso*-PGF_2α _production in microglia activated with LPS. Interestingly, two structurally different highly selective COX-1 inhibitors (SC-560 and VAS) potently reduced PGE_2 _production (Fig. 6A and 6C) and free radical formation (Fig. 6B and 6D) in activated microglia in a dose-dependent manner.

**Figure 7:**
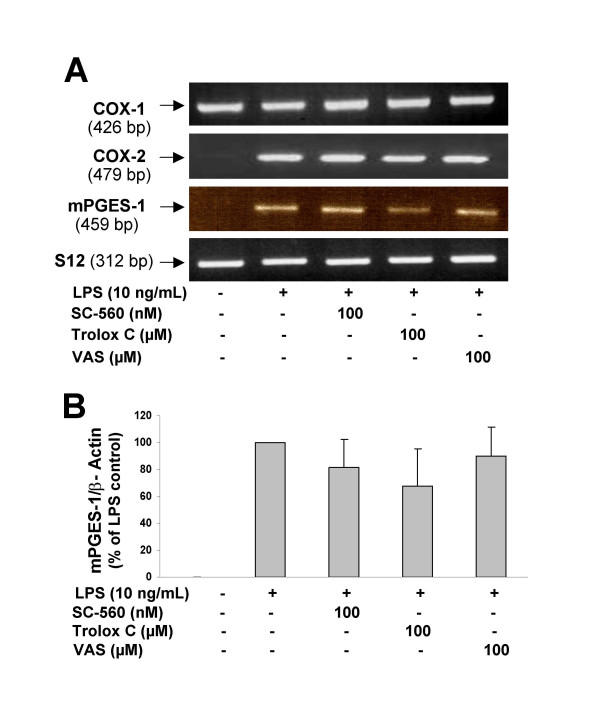
**A: **Effects of the antioxidant Trolox C and the COX-1 inhibitors (SC-560 and VAS) on the expression of COX-1, COX-2, and mPGES-1 mRNAs in rat microglial cells stimulated with LPS. RT-PCR analysis was performed after 4 h of incubation with LPS. There are no significant effects of the COX-1 inhibitors on mPGES-1 expression as demonstrated by the semi-quantitative analysis shown in Panel **B**. Trolox C produced a moderate reduction in mPGES-1 expression that did not attain statistical significance. Semi-quantitative analysis was done with results from 3 different RT-PCR experiments performed independently. Relative expression of rat S12 was used for normalization.

Next, we examined the potential effects of SC-560 and VAS on LPS-mediated mPGES-1 expression, which could help to explain if the effects of resveratrol on the reduction in mPGES-1 were mediated by a COX-1 mechanism. RT-PCR analysis reveals that COX-1 inhibition by SC-560 or VAS failed to reduce mPGES-1 expression in LPS-activated primary microglia (Fig. 7A and 7B).

Since previous studies have shown the ability of antioxidants to modify gene expression of pro-inflammatory mediators in activated microglia [31,45], we studied the effects of Trolox C on LPS-induced COX-2 and mPGES-1 expression. Trolox C slightly reduced mPGES-1 expression, although this effect did not reach statistical significance, as found in the densitometric analysis of three independent experiments (Fig. 7B). In another control experiment, we investigated the effects of COX-1 inhibitors and Trolox C on the expression of both COX isozymes. No effect of these compounds was observed in our *in vitro *model, as shown in Fig. 7. Furthermore, we also investigated if other COX inhibitors might have an effect on the expression of mPGES-1 by activated microglia. These compounds included the non-selective COX inhibitors indomethacin and ibuprofen, as well as the highly selective COX-2 inhibitor SC-58125. None of these compounds modify the LPS-induced mPGES-1 expression (data not shown).

## Discussion

Scientific literature on natural compounds has been recently accumulating an enormous amount of reports describing the neuroprotective ability of natural polyphenolic substances in different models of brain injury both *in vitro *and *in vivo*. Resveratrol is one of the most promising compounds, considering recent evidence indicating its potent ability to reduce cerebral damage after ischemia/hypoxia [7,13,17], trauma [18], excitotoxicity [8], and other conditions leading to neuronal demise [19,21].

It is widely accepted that neuroinflammation is a key player in various pathological events associated with brain injury. More specifically, microglial activation and the subsequent release of pro-inflammatory cytokines, ROS, and prostaglandins play a role of paramount importance in cerebral damage. Inhibition of COX-2 induction and/or activity has been proven to reduce brain injury after ischemia [46-50], excitotoxicity [47,51], and MPTP-induced neurodegeneration [52-54]. The most important mechanisms associated with the toxic effects of enhanced COX activity during neuroinflammation include production of PGE_2 _[55-59] and formation of free radicals leading to oxidative stress [34,37,60,61].

In the present study, we have found that resveratrol is a potent inhibitor of PGE_2 _and free radical formation by activated microglial cells. These findings add significant information on the molecular mechanisms involved in the neuroprotective effect of this compound. The ability of resveratrol to reduce PGE_2 _production comes from the modulation of multiple events in the COX/PGE_2 _pathway: 1) resveratrol is a potent inactivator of the peroxidase reaction of COX-1 [43], and thus is considered a relatively selective inhibitor of this isozyme; 2) resveratrol significantly diminished LPS-induced expression of mPGES-1 (Figs. 3 and 4), the most important terminal synthase responsible for PGE_2 _synthesis in activated microglia [42]; and 3) production of 8-*iso*-PGF_2α_, a reliable indicator of free radical generation, is dramatically reduced by low concentrations of resveratrol (Fig. 5).

The relative contribution of each of these mechanisms to the overall reduction in PGE_2 _by resveratrol is difficult to address based on present data. However, some issues deserve further discussion. The ability of resveratrol to inhibit the peroxidase activity of COX-1 is a well-known pharmacological effect of this compound [43,44]. COX-1 is constitutively expressed in microglia under resting conditions, and its expression is not induced by LPS as shown in Figs. 3A and 4A, and reported by us before [34]. However, according to present results, there is a significant contribution of COX-1 to PGE_2 _formation by microglia upon LPS challenge. This is supported by a previous study performed in LPS-stimulated human adult microglial cells, in which selective inhibition of COX-1 was also very effective in reducing PGE_2 _production [62]. Results from control experiments using other highly selective COX-1 inhibitors (SC-560 and VAS), in addition to resveratrol, indicate that COX-1 isoform is not only important in LPS-induced PGE_2 _synthesis, but it is also a key source of free radicals in microglia. This is an unexpected observation, and represents the first evidence that microglial COX-1 activity is a significant source of free radicals during neuroinflammation.

It has been previously shown that increased COX activity is associated with oxidative damage following different types of brain injury, including excitotoxicity [61,63,64], ischemia [60,65,66], and traumatic brain injury [67]. Furthermore, treatment with COX inhibitors has been proven to significantly reduce IL-1β- and LPS-induced oxidative damage in neuronal and microglial cells, respectively [34,37].

The interesting pharmacological properties of resveratrol in terms of inhibition of COX-1 and direct antioxidant ability, may underlie the dramatic attenuation of PGE_2 _and free radical production by LPS-activated microglia. It has long been known that free radicals directly increase COX activity and conversely antioxidants reduce COX catalytic activity [68-70]. Based on these reports, one may speculate that maintenance of microglial redox status by resveratrol contributes to the reduction in COX activity and PGE_2 _production in these cells. This is further supported by our finding that other antioxidants (Trolox C and α-tocopherol) are also able to reduce PGE_2 _formation (Fig. 1B). Antioxidants have been shown to modulate microglial activation [71-74]. In an earlier study, vitamin E was found to attenuate nitric oxide production and the induction of IL-1α and TNFα expression through suppression of signaling events necessary for microglial activation [71]. Furthermore, inhibition of ROS generation in LPS-activated microglia can reduce PGE_2 _production as reported previously by Wang et al [73].

An important new observation of the present study is the dramatic reduction in LPS-mediated expression of mPGES-1 in cells treated with resveratrol (Figs. 3 and 4). Based on very recent and convincing data, production of PGE_2 _in microglia following LPS treatment is almost entirely dependent on the activity of mPGES-1 [42]. Thus, blockade of mPGES-1 expression by resveratrol is an additional effect of this compound that contributes to its potent ability to block PGE_2 _synthesis in microglia. Resveratrol effects on mPGES-1 expression seem to be independent on its ability to reduce COX activity/PGE_2 _formation since other COX inhibitors were unable to modify LPS-induced mPGES-1 upregulation in microglial cells despite their potent inhibitory effects on PGE_2 _production (Figs. 6 and 7).

To the best of our knowledge, the present study is the first to document the ability of resveratrol to reduce mPGES-1 expression, as shown here in activated microglia. It is of great relevance that resveratrol reduced mPGES-1, but not COX-2 expression (Fig. 4). This suggests that LPS-induced microglial expression of mPGES-1 proceeds through molecular mechanisms which are different from the ones involved in COX-2 induction, providing for the first time evidence that the expression of mPGES-1 and COX-2 are not always coupled as suggested by other authors [75,76].

It has been recently reported that resveratrol reduces COX-2 expression in mouse BV-2 microglial cells through a mechanism that possibly involves inhibition of NF-κB activation [77]. Our present findings are not in line with these previous observations. The reasons for these discrepancies are not known, but might be related to different cell types and species (mouse BV-2 *versus *primary rat microglia).

At this point we have not yet identified the exact signal transduction pathway involved in resveratrol's effects on mPGES-1 expression. However, present findings will inspire new investigations in order to elucidate the differential signal transduction pathways responsible for the expression of mPGES-1 and COX-2 in microglia. This is of importance since this could lead to the discovery of new targets for attenuating microglial activation and PGE_2 _synthesis.

Because of the downstream position of mPGES-1 in the PGE_2_-synthesizing cascade, selective pharmacological blockade of its expression, as shown here for resveratrol, would affect only the pro-inflammatory PGE_2_, and would not decrease the production of other physiologically important prostanoids. Although COX-2 inhibitors have been highly marketed in the last five years including clinical trials in Alzheimer's disease, there are also important side effects associated with this new group of drugs [78,79]. Thus, it is of paramount importance our finding that resveratrol specifically reduced mPGES-1 without affecting COX-2 levels. Recent evidence indicates that enhanced expression and activity of mPGES-1 is a critical pathological event during inflammation, both in the CNS and in the periphery. Mice lacking the mPGES-1 gene are protected against stroke-induced injury [80] and display reduced pain hypersensitivity and inflammation [81,82].

## Conclusion

In summary, we are proposing here that significant attenuation of PGE_2 _and free radical production by activated microglia might contribute to the neuroprotective effects of resveratrol. This study gives further support to the potential use of resveratrol as a therapeutic agent to reduce microglial activation following different types of brain injury.

## Abbreviations

LPS (lipopolysaccharide); ROS (reactive oxygen species); PGE_2 _(prostaglandin E_2_); COX (cyclooxygenase); mPGES-1 (microsomal prostaglandin E synthase-1); EIA (enzyme immunoassay); DTT (1,4-Dithio-DL-threitol); BSA (bovine serum albumin); ANOVA (analysis of variance); 8-*iso-*PGF_2α _(8-*iso*-prostaglandin F_2α_); VAS (Valeroyl Salicylate).

## Competing interests

The author(s) declare that they have no competing interests.

## Authors' contributions

ECJ designed the study, performed 8-*iso*-PGF_2α _assay, Western blot and RT-PCR analysis, partly directed the work, reviewed the data, and wrote the manuscript. ACPO, SG and HSB performed Western blot and RT-PCR analysis. MH and EM provided consultation and reviewed the data. BLF directed the work, contributed to the design of the study, reviewed the data and helped to write the manuscript. All authors read and approved the final manuscript.

## References

[B1] Soleas GJ, Diamandis EP, Goldberg DM (1997). Resveratrol: a molecule whose time has come? And gone?. Clin Biochem.

[B2] Fremont L (2000). Biological effects of resveratrol. Life Sci.

[B3] Gusman J, Malonne H, Atassi G (2001). A reappraisal of the potential chemopreventive and chemotherapeutic properties of resveratrol. Carcinogenesis.

[B4] Gao X, Xu YX, Janakiraman N, Chapman RA, Gautam SC (2001). Immunomodulatory activity of resveratrol: suppression of lymphocyte proliferation, development of cell-mediated cytotoxicity, and cytokine production. Biochem Pharmacol.

[B5] Gao X, Deeb D, Media J, Divine G, Jiang H, Chapman RA, Gautam SC (2003). Immunomodulatory activity of resveratrol: discrepant in vitro and in vivo immunological effects. Biochem Pharmacol.

[B6] Goh SS, Woodman OL, Pepe S, Cao AH, Qin C, Ritchie RH (2007). The red wine antioxidant resveratrol prevents cardiomyocyte injury following ischemia-reperfusion via multiple sites and mechanisms. Antioxid Redox Signal.

[B7] Wang Q, Xu J, Rottinghaus GE, Simonyi A, Lubahn D, Sun GY, Sun AY (2002). Resveratrol protects against global cerebral ischemic injury in gerbils. Brain Res.

[B8] Wang Q, Yu S, Simonyi A, Rottinghaus G, Sun GY, Sun AY (2004). Resveratrol protects against neurotoxicity induced by kainic acid. Neurochem Res.

[B9] Ates O, Cayli SR, Yucel N, Altinoz E, Kocak A, Durak MA, Turkoz Y, Yologlu S (2007). Central nervous system protection by resveratrol in streptozotocin-induced diabetic rats. J Clin Neurosci.

[B10] Zhuang H, Kim YS, Koehler RC, Dore S (2003). Potential mechanism by which resveratrol, a red wine constituent, protects neurons. Ann N Y Acad Sci.

[B11] Bastianetto S, Zheng WH, Quirion R (2000). Neuroprotective abilities of resveratrol and other red wine constituents against nitric oxide-related toxicity in cultured hippocampal neurons. Br J Pharmacol.

[B12] Han YS, Zheng WH, Bastianetto S, Chabot JG, Quirion R (2004). Neuroprotective effects of resveratrol against beta-amyloid-induced neurotoxicity in rat hippocampal neurons: involvement of protein kinase C. Br J Pharmacol.

[B13] Huang SS, Tsai MC, Chih CL, Hung LM, Tsai SK (2001). Resveratrol reduction of infarct size in Long-Evans rats subjected to focal cerebral ischemia. Life Sci.

[B14] Tsai SK, Hung LM, Fu YT, Cheng H, Nien MW, Liu HY, Zhang FB, Huang SS (2007). Resveratrol neuroprotective effects during focal cerebral ischemia injury via nitric oxide mechanism in rats. J Vasc Surg.

[B15] Mokni M, Elkahoui S, Limam F, Amri M, Aouani E (2007). Effect of resveratrol on antioxidant enzyme activities in the brain of healthy rat. Neurochem Res.

[B16] Lu KT, Chiou RY, Chen LG, Chen MH, Tseng WT, Hsieh HT, Yang YL (2006). Neuroprotective effects of resveratrol on cerebral ischemia-induced neuron loss mediated by free radical scavenging and cerebral blood flow elevation. J Agric Food Chem.

[B17] Zamin LL, llenburg-Pilla P, Argenta-Comiran R, Horn AP, Simao F, Nassif M, Gerhardt D, Frozza RL, Salbego C (2006). Protective effect of resveratrol against oxygen-glucose deprivation in organotypic hippocampal slice cultures: Involvement of PI3-K pathway. Neurobiol Dis.

[B18] Ates O, Cayli S, Altinoz E, Gurses I, Yucel N, Sener M, Kocak A, Yologlu S (2007). Neuroprotection by resveratrol against traumatic brain injury in rats. Mol Cell Biochem.

[B19] Kiziltepe U, Turan NN, Han U, Ulus AT, Akar F (2004). Resveratrol, a red wine polyphenol, protects spinal cord from ischemia-reperfusion injury. J Vasc Surg.

[B20] Ates O, Cayli S, Altinoz E, Gurses I, Yucel N, Kocak A, Yologlu S, Turkoz Y (2006). Effects of resveratrol and methylprednisolone on biochemical, neurobehavioral and histopathological recovery after experimental spinal cord injury. Acta Pharmacol Sin.

[B21] Kumar P, Padi SS, Naidu PS, Kumar A (2006). Effect of resveratrol on 3-nitropropionic acid-induced biochemical and behavioural changes: possible neuroprotective mechanisms. Behav Pharmacol.

[B22] Marambaud P, Zhao H, Davies P (2005). Resveratrol promotes clearance of Alzheimer's disease amyloid-beta peptides. J Biol Chem.

[B23] Streit WJ, Mrak RE, Griffin WS (2004). Microglia and neuroinflammation: a pathological perspective. J Neuroinflammation.

[B24] Kim SU, de Vellis J (2005). Microglia in health and disease. J Neurosci Res.

[B25] Minghetti L (2005). Role of inflammation in neurodegenerative diseases. Curr Opin Neurol.

[B26] Li Y, Liu L, Barger SW, Griffin WS (2003). Interleukin-1 mediates pathological effects of microglia on tau phosphorylation and on synaptophysin synthesis in cortical neurons through a p38-MAPK pathway. J Neurosci.

[B27] Block ML, Hong JS (2005). Microglia and inflammation-mediated neurodegeneration: multiple triggers with a common mechanism. Prog Neurobiol.

[B28] Block ML, Zecca L, Hong JS (2007). Microglia-mediated neurotoxicity: uncovering the molecular mechanisms. Nat Rev Neurosci.

[B29] Bi XL, Yang JY, Dong YX, Wang JM, Cui YH, Ikeshima T, Zhao YQ, Wu CF (2005). Resveratrol inhibits nitric oxide and TNF-alpha production by lipopolysaccharide-activated microglia. Int Immunopharmacol.

[B30] Fiebich BL, Biber K, Lieb K, van CD, Berger M, Bauer J, Gebicke-Haerter PJ (1996). Cyclooxygenase-2 expression in rat microglia is induced by adenosine A2a-receptors. Glia.

[B31] Bauer MK, Lieb K, Schulze-Osthoff K, Berger M, Gebicke-Haerter PJ, Bauer J, Fiebich BL (1997). Expression and regulation of cyclooxygenase-2 in rat microglia. Eur J Biochem.

[B32] Fiebich BL, Lieb K, Hull M, Aicher B, van RJ, Pairet M, Engelhardt G (2000). Effects of caffeine and paracetamol alone or in combination with acetylsalicylic acid on prostaglandin E(2) synthesis in rat microglial cells. Neuropharmacology.

[B33] Lieb K, Engels S, Fiebich BL (2003). Inhibition of LPS-induced iNOS and NO synthesis in primary rat microglial cells. Neurochem Int.

[B34] Akundi RS, Candelario-Jalil E, Hess S, Hull M, Lieb K, Gebicke-Haerter PJ, Fiebich BL (2005). Signal transduction pathways regulating cyclooxygenase-2 in lipopolysaccharide-activated primary rat microglia. Glia.

[B35] Gebicke-Haerter PJ, Bauer J, Schobert A, Northoff H (1989). Lipopolysaccharide-free conditions in primary astrocyte cultures allow growth and isolation of microglial cells. J Neurosci.

[B36] Fiebich BL, Lieb K, Kammerer N, Hull M (2003). Synergistic inhibitory effect of ascorbic acid and acetylsalicylic acid on prostaglandin E2 release in primary rat microglia. J Neurochem.

[B37] Candelario-Jalil E, Akundi RS, Bhatia HS, Lieb K, Appel K, Munoz E, Hull M, Fiebich BL (2006). Ascorbic acid enhances the inhibitory effect of aspirin on neuronal cyclooxygenase-2-mediated prostaglandin E(2) production. J Neuroimmunol.

[B38] Chomczynski P, Sacchi N (1987). Single-step method of RNA isolation by acid guanidinium thiocyanate-phenol-chloroform extraction. Anal Biochem.

[B39] (2007). Primer3 Software. http://frodo.wi.mit.edu/primer3/input.htm.

[B40] Laemmli UK (1970). Cleavage of structural proteins during the assembly of the head of bacteriophage T4. Nature.

[B41] Pratico D, Barry OP, Lawson JA, Adiyaman M, Hwang SW, Khanapure SP, Iuliano L, Rokach J, FitzGerald GA (1998). IPF2alpha-I: an index of lipid peroxidation in humans. Proc Natl Acad Sci U S A.

[B42] Ikeda-Matsuo Y, Ikegaya Y, Matsuki N, Uematsu S, Akira S, Sasaki Y (2005). Microglia-specific expression of microsomal prostaglandin E2 synthase-1 contributes to lipopolysaccharide-induced prostaglandin E2 production. J Neurochem.

[B43] Szewczuk LM, Forti L, Stivala LA, Penning TM (2004). Resveratrol is a peroxidase-mediated inactivator of COX-1 but not COX-2: a mechanistic approach to the design of COX-1 selective agents. J Biol Chem.

[B44] Kummerle AE, Sperandio da Silva GM, Sant'Anna CM, Barreiro EJ, Fraga CA (2005). A proposed molecular basis for the selective resveratrol inhibition of the PGHS-1 peroxidase activity. Bioorg Med Chem.

[B45] Egger T, Schuligoi R, Wintersperger A, Amann R, Malle E, Sattler W (2003). Vitamin E (alpha-tocopherol) attenuates cyclo-oxygenase 2 transcription and synthesis in immortalized murine BV-2 microglia. Biochem J.

[B46] Nogawa S, Zhang F, Ross ME, Iadecola C (1997). Cyclo-oxygenase-2 gene expression in neurons contributes to ischemic brain damage. J Neurosci.

[B47] Iadecola C, Niwa K, Nogawa S, Zhao X, Nagayama M, Araki E, Morham S, Ross ME (2001). Reduced susceptibility to ischemic brain injury and N-methyl-D-aspartate-mediated neurotoxicity in cyclooxygenase-2-deficient mice. Proc Natl Acad Sci U S A.

[B48] Candelario-Jalil E, Mhadu NH, Gonzalez-Falcon A, Garcia-Cabrera M, Munoz E, Leon OS, Fiebich BL (2005). Effects of the cyclooxygenase-2 inhibitor nimesulide on cerebral infarction and neurological deficits induced by permanent middle cerebral artery occlusion in the rat. J Neuroinflammation.

[B49] Candelario-Jalil E, Alvarez D, Gonzalez-Falcon A, Garcia-Cabrera M, Martinez-Sanchez G, Merino N, Giuliani A, Leon OS (2002). Neuroprotective efficacy of nimesulide against hippocampal neuronal damage following transient forebrain ischemia. Eur J Pharmacol.

[B50] Dore S, Otsuka T, Mito T, Sugo N, Hand T, Wu L, Hurn PD, Traystman RJ, Andreasson K (2003). Neuronal overexpression of cyclooxygenase-2 increases cerebral infarction. Ann Neurol.

[B51] Kelley KA, Ho L, Winger D, Freire-Moar J, Borelli CB, Aisen PS, Pasinetti GM (1999). Potentiation of excitotoxicity in transgenic mice overexpressing neuronal cyclooxygenase-2. Am J Pathol.

[B52] Teismann P, Tieu K, Choi DK, Wu DC, Naini A, Hunot S, Vila M, Jackson-Lewis V, Przedborski S (2003). Cyclooxygenase-2 is instrumental in Parkinson's disease neurodegeneration. Proc Natl Acad Sci U S A.

[B53] Hunot S, Vila M, Teismann P, Davis RJ, Hirsch EC, Przedborski S, Rakic P, Flavell RA (2004). JNK-mediated induction of cyclooxygenase 2 is required for neurodegeneration in a mouse model of Parkinson's disease. Proc Natl Acad Sci U S A.

[B54] Vijitruth R, Liu M, Choi DY, Nguyen XV, Hunter RL, Bing G (2006). Cyclooxygenase-2 mediates microglial activation and secondary dopaminergic cell death in the mouse MPTP model of Parkinson's disease. J Neuroinflammation.

[B55] Minghetti L, Levi G (1998). Microglia as effector cells in brain damage and repair: focus on prostanoids and nitric oxide. Prog Neurobiol.

[B56] Kawano T, Anrather J, Zhou P, Park L, Wang G, Frys KA, Kunz A, Cho S, Orio M, Iadecola C (2006). Prostaglandin E2 EP1 receptors: downstream effectors of COX-2 neurotoxicity. Nat Med.

[B57] Phillis JW, Horrocks LA, Farooqui AA (2006). Cyclooxygenases, lipoxygenases, and epoxygenases in CNS: their role and involvement in neurological disorders. Brain Res Rev.

[B58] Candelario-Jalil E, Gonzalez-Falcon A, Garcia-Cabrera M, Leon OS, Fiebich BL (2007). Post-ischaemic treatment with the cyclooxygenase-2 inhibitor nimesulide reduces blood-brain barrier disruption and leukocyte infiltration following transient focal cerebral ischaemia in rats. J Neurochem.

[B59] Candelario-Jalil E, Gonzalez-Falcon A, Garcia-Cabrera M, Leon OS, Fiebich BL (2004). Wide therapeutic time window for nimesulide neuroprotection in a model of transient focal cerebral ischemia in the rat. Brain Res.

[B60] Candelario-Jalil E, Gonzalez-Falcon A, Garcia-Cabrera M, Alvarez D, Al-Dalain S, Martinez G, Leon OS, Springer JE (2003). Assessment of the relative contribution of COX-1 and COX-2 isoforms to ischemia-induced oxidative damage and neurodegeneration following transient global cerebral ischemia. J Neurochem.

[B61] Pepicelli O, Fedele E, Berardi M, Raiteri M, Levi G, Greco A, Ajmone-Cat MA, Minghetti L (2005). Cyclo-oxygenase-1 and -2 differently contribute to prostaglandin E2 synthesis and lipid peroxidation after in vivo activation of N-methyl-D-aspartate receptors in rat hippocampus. J Neurochem.

[B62] Hoozemans JJ, Veerhuis R, Janssen I, van Elk EJ, Rozemuller AJ, Eikelenboom P (2002). The role of cyclo-oxygenase 1 and 2 activity in prostaglandin E(2) secretion by cultured human adult microglia: implications for Alzheimer's disease. Brain Res.

[B63] Candelario-Jalil E, Ajamieh HH, Sam S, Martinez G, Leon Fernandez OS (2000). Nimesulide limits kainate-induced oxidative damage in the rat hippocampus. Eur J Pharmacol.

[B64] Pepicelli O, Fedele E, Bonanno G, Raiteri M, Ajmone-Cat MA, Greco A, Levi G, Minghetti L (2002). In vivo activation of N-methyl-D-aspartate receptors in the rat hippocampus increases prostaglandin E(2) extracellular levels and triggers lipid peroxidation through cyclooxygenase-mediated mechanisms. J Neurochem.

[B65] Candelario-Jalil E, Alvarez D, Merino N, Leon OS (2003). Delayed treatment with nimesulide reduces measures of oxidative stress following global ischemic brain injury in gerbils. Neurosci Res.

[B66] Miyamoto O, Tamae K, Kasai H, Hirakawa H, Hayashida Y, Konishi R, Itano T (2003). Suppression of hyperemia and DNA oxidation by indomethacin in cerebral ischemia. Eur J Pharmacol.

[B67] Tyurin VA, Tyurina YY, Borisenko GG, Sokolova TV, Ritov VB, Quinn PJ, Rose M, Kochanek P, Graham SH, Kagan VE (2000). Oxidative stress following traumatic brain injury in rats: quantitation of biomarkers and detection of free radical intermediates. J Neurochem.

[B68] Vanderhoek JY, Lands WE (1973). The inhibition of the fatty acid oxygenase of sheep vesicular gland by antioxidants. Biochim Biophys Acta.

[B69] Hemler ME, Lands WE (1980). Evidence for a peroxide-initiated free radical mechanism of prostaglandin biosynthesis. J Biol Chem.

[B70] Beharka AA, Wu D, Serafini M, Meydani SN (2002). Mechanism of vitamin E inhibition of cyclooxygenase activity in macrophages from old mice: role of peroxynitrite. Free Radic Biol Med.

[B71] Li Y, Liu L, Barger SW, Mrak RE, Griffin WS (2001). Vitamin E suppression of microglial activation is neuroprotective. J Neurosci Res.

[B72] Pawate S, Shen Q, Fan F, Bhat NR (2004). Redox regulation of glial inflammatory response to lipopolysaccharide and interferongamma. J Neurosci Res.

[B73] Wang T, Qin L, Liu B, Liu Y, Wilson B, Eling TE, Langenbach R, Taniura S, Hong JS (2004). Role of reactive oxygen species in LPS-induced production of prostaglandin E2 in microglia. J Neurochem.

[B74] Hsiao G, Fong TH, Tzu NH, Lin KH, Chou DS, Sheu JR (2004). A potent antioxidant, lycopene, affords neuroprotection against microglia activation and focal cerebral ischemia in rats. In Vivo.

[B75] Murakami M, Naraba H, Tanioka T, Semmyo N, Nakatani Y, Kojima F, Ikeda T, Fueki M, Ueno A, Oh S, Kudo I (2000). Regulation of prostaglandin E2 biosynthesis by inducible membrane-associated prostaglandin E2 synthase that acts in concert with cyclooxygenase-2. J Biol Chem.

[B76] Kudo I, Murakami M (2005). Prostaglandin E synthase, a terminal enzyme for prostaglandin E2 biosynthesis. J Biochem Mol Biol.

[B77] Heynekamp JJ, Weber WM, Hunsaker LA, Gonzales AM, Orlando RA, Deck LM, Jagt DL (2006). Substituted trans-stilbenes, including analogues of the natural product resveratrol, inhibit the human tumor necrosis factor alpha-induced activation of transcription factor nuclear factor kappaB. J Med Chem.

[B78] Crofford LJ (2000). Clinical experience with specific COX-2 inhibitors in arthritis. Curr Pharm Des.

[B79] Crofford LJ, Breyer MD, Strand CV, Rushitzka F, Brune K, Farkouh ME, Simon LS (2006). Cardiovascular effects of selective COX-2 inhibition: is there a class effect? The International COX-2 Study Group. J Rheumatol.

[B80] Ikeda-Matsuo Y, Ota A, Fukada T, Uematsu S, Akira S, Sasaki Y (2006). Microsomal prostaglandin E synthase-1 is a critical factor of stroke-reperfusion injury. Proc Natl Acad Sci U S A.

[B81] Kamei D, Yamakawa K, Takegoshi Y, Mikami-Nakanishi M, Nakatani Y, Oh-Ishi S, Yasui H, Azuma Y, Hirasawa N, Ohuchi K, Kawaguchi H, Ishikawa Y, Ishii T, Uematsu S, Akira S, Murakami M, Kudo I (2004). Reduced pain hypersensitivity and inflammation in mice lacking microsomal prostaglandin e synthase-1. J Biol Chem.

[B82] Trebino CE, Stock JL, Gibbons CP, Naiman BM, Wachtmann TS, Umland JP, Pandher K, Lapointe JM, Saha S, Roach ML, Carter D, Thomas NA, Durtschi BA, McNeish JD, Hambor JE, Jakobsson PJ, Carty TJ, Perez JR, Audoly LP (2003). Impaired inflammatory and pain responses in mice lacking an inducible prostaglandin E synthase. Proc Natl Acad Sci U S A.

